# Digital media exposure and cognitive functioning in European children and adolescents of the I.Family study

**DOI:** 10.1038/s41598-023-45944-0

**Published:** 2023-11-01

**Authors:** Elida Sina, Christoph Buck, Wolfgang Ahrens, Juul M. J. Coumans, Gabriele Eiben, Annarita Formisano, Lauren Lissner, Artur Mazur, Nathalie Michels, Dénes Molnar, Luis A. Moreno, Valeria Pala, Hermann Pohlabeln, Lucia Reisch, Michael Tornaritis, Toomas Veidebaum, Antje Hebestreit, I. Family consortium

**Affiliations:** 1 Leibniz Institute for Prevention Research and Epidemiology - BIPS, Bremen, Germany; 2 Faculty of Mathematics and Computer Science, Institute of Statistics, University of Bremen, Bremen, Germany; 3 Teaching and Learning Centre, Open University of the Netherlands, Heerlen, The Netherlands; 4 Department of Public Health, School of Health Sciences, University of Skövde, Skövde, Sweden; 5 Institute of Food Sciences, National Research Council, Avellino, Italy; 6 School of Public Health and Community Medicine, Institute of Medicine, Sahlgrenska Academy, University of Gothenburg, Gothenburg, Sweden; 7 Faculty of Medicine, University of Rzeszów, Rzeszów, Poland; 8 Department of Public Health and Primary Care, Ghent University, Ghent, Belgium; 9 Department of Pediatrics, Medical School, University of Pécs, Pécs, Hungary; 10 GENUD (Growth, Exercise, Nutrition and Development) Research Group, Centro de Investigación Biomédica en Red Fisiopatología de La Obesidad y Nutrición (CIBERObn), Instituto Agroalimentario de Aragón (IA2), Instituto de Investigación Sanitaria Aragón (IIS Aragón), University of Zaragoza, Zaragoza, Spain; 11 Department of Epidemiology and Data Science, Fondazione IRCCS, Istituto Nazionale Dei Tumori, Milan, Italy; 12 Cambridge Judge Business School, University of Cambridge, Cambridge, UK; 13 Research and Education Institute of Child Health, Strovolos, Cyprus; 14 Department of Chronic Diseases, National Institute for Health Development, Tallinn, Estonia

**Keywords:** Epidemiology, Risk factors, Cognitive control, Decision, Personality

## Abstract

The digital environment can pose health risks through exposure to unhealthy content. Yet, little is known about its relation to children’s cognitive functioning**.** This study investigates the association between digital media (DM) exposure and children’s cognitive functioning. This cross-sectional study is based on examinations of children aged 8–18 years (N = 8673) of the I.Family cohort (2013–2014). Exposure to television, computer, smartphone and internet was self-reported (hours/day). Media multitasking (MMT) was defined as simultaneous use of computers with other digital or non-screen-based activities. Standard instruments were used to assess cognitive inflexibility (score: 0–39), decision-making ability (− 100 to + 100) and impulsivity (12–48). Adjusted regression coefficients and 99.9%CIs were calculated by generalized linear mixed-effects models. In total, 3261 participants provided data for impulsivity, 3441 for cognitive inflexibility and 4046 for decision-making. Exposure to smartphones and media multitasking were positively associated with impulsivity (β_smartphone_ = 0.74; 99.9%CI = 0.42–1.07; β_MMT_ = 0.73; 99.9%CI = 0.35–1.12) and cognitive inflexibility (β_smartphone_ = 0.32; 99.9%CI = -0.02–0.66; β_MMT_ = 0.39; 99.9%CI = 0.01–0.77) while being inversely associated with decision-making ability. Extensive smartphone/internet exposure combined with low computer/medium TV exposure was associated with higher impulsivity and cognitive inflexibility scores, especially in girls. DM exposure is adversely associated with cognitive functioning in children and adolescents. Children require protection against the likely adverse impact of digital environment.

Today’s children and adolescents are growing up in a digital media (DM) saturated environment, and they increasingly spend time with televisions, computers, video-games and smartphones. In the US, children and adolescents use DM for entertainment for five and eight hours daily, respectively, more than any other waking activity^1^. European children aged 9–16 years use online media for almost three hours/day^2^. Hence, DM represents a fundamental part of the environment in which children grow up. Therefore, it is crucial to investigate the role of DM exposure on children’s health.

It is well-documented that DM exposure is positively associated with unhealthy dietary patterns during childhood and adolescence^3–5^, and obesity in adulthood^6–9^. Moreover, studies have shed light on the deleterious role of DM exposure on children’s and adolescents’ psychosocial well-being^10,11^ and body image^12^. The impact of DM exposure seems to extend beyond obesity and well-being, by influencing children’s cognitive development as well^13^. In fact, children’s brain and neural structure and their cognitive functioning are shaped through interactions with the external environment, including the digital environment^14^. Nowadays, DM is intertwined with children’s lives, and an excessive exposure to DM during childhood, when the brain is highly plastic, might deteriorate the healthy development of brain structures. Studies conducted in laboratory conditions using functional magnetic resonance imaging (fMRI) have shown that prolonged exposure to screen-based media is associated with reduced microstructural integrity of the brain white matter in areas related to language, attention, and executive functioning in children^15^ and adolescents^16^. Sound cognitive functioning is important for making healthy lifestyle choices, known as neuro-selection. Children with poor cognitive functioning are more likely to engage in unhealthy behaviours, such as consumption of unhealthy foods^17^, but also smoking and alcohol drinking later in adulthood^18^.

Non-educational television viewing (TV) has been associated with reduced language skills and executive functioning among pre-schoolers due to exposure to adult-directed programmes and reduced parent–child interactions^19^. A recent meta-analysis showed that DM exposure (TV and video-gaming) negatively impacted the academic performance of children aged 4–18 years^20^. This impact is also observed for smartphones^21^, which facilitate the ubiquitous access to internet, messaging applications and social media (SM). The prolific information provided by DM, the urge to constantly check notifications and online content, may lead to over-stimulation and impact children’s emotion regulation, distract them during routine tasks, limiting their cognitive processing capacities^22,23^. Furthermore, laboratory based studies have shown that excessive smartphone use among adolescents is associated with lower connectivity in the prefrontal cortex and the anterior cingulate region of the brain, which are specialized in inhibition control (i.e., impulsivity) and cognitive flexibility, respectively^24^. These latter constructs were previously positively associated with unhealthy snack consumption^25^ and unfavourable weight status among adolescents^26^. Media multitasking (MMT) is a common behaviour among today’s youth and refers to using multiple media devices simultaneously (e.g., PC used while watching TV) or using DM while engaged in non-media activities (e.g., PC used while reading a book). MMT has been associated with cognitive outcomes, including long-term attention problems^27^, poor memory and reduced volume in anterior cingulate cortex, a region implicated in cognitive and socio-emotional control^28^.

Given the limited empirical evidence on the role of digital environment on children’s cognitive functioning, further research outside laboratory conditions is required. Therefore, this study investigates the association between DM exposure, including smartphone, PC, TV and internet use as well as MMT, on several measures of cognition – namely emotion-driven impulsiveness, decision-making ability and cognitive inflexibility – in a sample of European children and adolescents aged 8 to 18 years, in free-living conditions. We consider differences in the family environment, such as parental education ^13^ and family structure in the abovementioned associations. Moreover, we use a latent class analysis to identify underlying patterns of DM use based on the examined single media exposures to better understand the impact of DM exposure on children’s cognitive functioning.

## Methods

### Study design and setting

This cross-sectional study exploits data from the I.Family study (2013—2014) conducted across nine countries, i.e., Belgium, Estonia, Cyprus, Hungary, Italy, Germany, Poland, Spain and Sweden, following standardized instruments and protocols^29^. Across study centres, we included children aged ≥ 8 years who provided information on three distinct measures of cognitive functioning: (i) emotion-driven impulsiveness, (ii) decision-making ability, and (iii) cognitive inflexibility. Besides age, primary exclusion criteria were implausible self-reports on DM use or a self-reported medical ADHD-diagnosis (Supplementary Fig. S1, Supplementary Methods). Adolescents and parents of all children provided written informed consent. Children (< 12 years) provided their oral assent. All procedures followed were in accordance with the ethical standards of the Helsinki Declaration of 1975, and its later amendments. Ethical approval was obtained from local institutional review boards at each study centre: (1) Belgium: Ethics Committee of the Gent University Hospital, 15/10/2007, ref: no. EC UZG 2007/243 and 19/02/2013, No. B670201316342; (2) Cyprus: Cyprus National Bioethics Committee, 12/07/2007, ref: no. EEBK/EM/2007/16 and 21/Feb/2013, No. EEBK/ETI/2012/33; (3) Estonia: Tallinn Medical Research Ethics Committee (TMREC), 14/06/2007, ref: no. 1093 and 17/January 2013, No. 128; (4) Germany: Ethic Commission of the University of Bremen, 16/01/2007 and 11/12/2012; (5) Hungary: Medical Research Council, 21/Jun/2007, ref: 22-156/2007-1018EKU and 18/12/2012, 4536/2013/EKU; (6) Italy: Ethics Committee of the Local Health Authority (ASL) in Avellino, 19/06/2007, ref: no. 2/CE and 18/Sep/2012, No. 12/12; (7) Spain: Ethics Committee for Clinical Research of Aragon (CEICA), 20/06/2007, ref:no. PI07/13 and 13/Feb/2013, No. PI13/0012; (8) Sweden: Regional Ethics Research Board in Gothenburg, 30/07/2007, ref: no. 264–07 and 10/Jan/2013, No. 927–12; (9) Poland: Bioethical Committee of the University of Rzeszów, 05/06/2013 and 01/12/2015.

### Data collection

#### Core questionnaire and assessment of media use

Age, sex, and country of residence were self-reported by adolescents (≥ 12 years) and proxy-reported by parents of younger children (< 12 years). Information on DM use duration, MMT, and confounding variables were measured using standardized questionnaires, previously tested for relative validity and reproducibility^30^. Participants reported the DM use duration separately for weekdays and weekend days, including TV/DVD/video, computer/game console (PC), and internet use (Supplementary Methods). In order to prevent a potential overlap with internet use, for PC use participants were asked “How long do you usually sit at a computer/game console per day? (Please disregard the time spent on internet use.)”, which enabled the assessment of off-line use of PC and game consoles. Total duration of TV, PC and internet use was respectively calculated as the sum of the weighted durations during weekdays and weekend days (hours/week), and quantified as hours/day. We measured smartphone use asking: “Thinking only about yesterday, about how much time did you spend watching TV shows, movies or music videos on a cell phone?”. On a 5-point Likert-scale, answers ranged from 0 (not at all) to 5 (> 3 h/day). An attributed time was assigned to each category to calculate the duration (hours/day) of smartphone use. Moreover, we measured MMT asking whether children engaged in other activities while using PCs, including TV, sending text messages, playing video-games, listening to music and reading. Based on dichotomized answers (“yes” or “no), a composite score of media multitasking behaviour ranging from 0 to 5 was calculated.

### Emotion-driven impulsiveness

To assess emotion-driven impulsiveness (EDI), 3400 children aged ≥ 8 years self-completed the 12-item negative urgency subscale from the Urgency, Premeditation, Perseverance, Sensation seeking, and Positive urgency (UPPS-P) questionnaire^31^. Participants rated items on a 4-point Likert scale ranging from 1 (agree strongly) to 4 (disagree strongly). All items were recoded except for one item, to make sure that all items ran in the same direction. For participants who completed all items (those with incomplete items were excluded), a sum score for EDI was calculated ranging from 12 to 48 ^31^. A higher score indicated higher impulsivity. After all exclusion criteria (incomplete items of the subscale in addition to primary exclusion criteria, Supplementary Fig. S1) were applied, 3261 children aged 9.9–17.9 years remained for the final analyses on impulsivity.

### Cognitive inflexibility

To measure cognitive inflexibility (CIF), 4034 children and adolescents performed a computerised version of the Berg Card Sorting test^32,33^. Four cards of different colours and shapes, and a deck consisting of 64 stimulus cards were shown to the participant. Participants had to sort cards according to a particular rule (by symbol, number or colour) that was unknown to them, by choosing one of the key cards (e.g., if ‘by colour’ is the correct rule, the colour of symbols on the stimulus card should match the colour of symbols on the key card). A feedback message (‘correct’ or ‘incorrect’) was provided to the participants after sorting each card. The rule was changed without notice after 10 consecutive correct trials, and the participant had to find out the new rule. The number of perseverative errors after the rule had changed, i.e., the number of cards sorted according to the previous rule, was used as the measure of CIF. A higher number of errors indicates higher CIF. After all exclusion criteria were applied (Supplementary Fig. S1), 3441 children aged 8–17.9 years remained in the final analysis group.

### Decision-making ability

Decision-making ability (DMA) was measured in 4169 children aged 8–18 years, using a computerised version of the Hungry Donkey Test^34^, the child-friendly version of the Iowa Gambling Task^35^, consisting of 100 trials. In each trial, participants should help a hungry donkey to collect apples by choosing one of the four doors presented on the screen. Each choice resulted in reward (apples) or in punishment (loss of apples). Doors 1&2 were disadvantageous doors because they yielded larger immediate reward but led to losing more apples in the long-term, resulting in net loss. Doors 3&4 were advantageous doors because they yielded smaller immediate rewards but led to winning more apples in the long-term, resulting in net gain^34^. DMA was calculated by subtracting the number of advantageous choices (doors 3&4) from the number of disadvantageous choices (doors 1&2), resulting in a score ranging from − 100 to + 100. A higher DMA is characterised by more advantageous choices than disadvantageous ones. After all exclusion criteria were applied (Supplementary Fig. S1), 4046 children aged 8–17.9 years remained for the final analyses on DMA.

### Potential confounders

A vast array of confounders was self- or proxy-reported via questionnaires. Besides age, sex and country of residence, we also included information on parental highest education attainment^36^, the number of media rules at home^37^, pubertal status^38,39^, weight status (objectively-measured)^40^, total daily sleep duration and psychosocial well-being^41^. Moreover, via a kinship and household interview^42^, parents reported on family structure, including whether the participating child was an only child in the household and whether he/she lived in a one-parent or a two-parent family. Detailed explanation on measurements, operationalization and the rationale of including the confounding variables is provided in Supplementary Methods.

### Statistical analyses

Response proportions differed for the cognitive tests and UPPS-P questionnaire resulting in three overlapping analysis groups (Supplementary Fig. S1), thus descriptive analyses were conducted separately for each cognitive outcome. Characteristics of age, sex, parental education attainment, weight status, pubertal status, family structure, country, MMT and media rules at home were tabulated for each analysis group. Children’s performance on the cognitive tests was also calculated. To account for missing values, standard fully conditional specification multiple imputation (MI) was performed with 10 replications. This procedure has demonstrated unbiased handling of missing values, and enables the inclusion of both continuous and categorical variables in the imputation model^43^. All exposures and covariates used in the analyses were included in the MI, except the outcomes due to high percentage of missing values (> 50%). The relative efficiency of imputation (i.e., how well the true population parameters are estimated) for all variables was ≥ 98%, indicating good imputation quality. The proportion of missing information ranged from 0.5% for weight status to 26% for puberty based on Tanner stages. The characteristics of imputed and non-imputed analysis groups are shown in Supplementary Table S1. To examine the role of DM exposure on cognitive outcomes, a two-step analysis approach was conducted:

Step 1: Association of single DM exposures with cognitive outcomes

The associations between duration of using single DM and single outcomes were examined using generalized linear mixed regressions, adjusting for confounders. To assess potential multicollinearity of DM variables, we included TV, PC, internet, smartphone, MMT and media rules in the same regression model (step two) and calculated the tolerance and variance inflation factor (our limit: < 10)^44^, which indicated lack of multicollinearity (data not shown).

Step 2: Latent profiles of DM use in association with cognitive outcomes

To identify underlying latent profiles of DM use, latent class analyses (LCA) were conducted^45^ with three categories of duration for each DM variable (low duration: < 1 h/day; medium duration: 1–2 h/day; high duration: > 2 h/day). LCA was performed using two to six latent profiles of four variables (TV, PC, smartphone and internet), clustered by country, as we previously observed country-differences on DM use^46^. Models were then compared based on the Bayesian Information Criterium (BIC) and a clear distinction of latent profiles in terms of conditional probabilities (Supplementary Table S2). The chosen profiles (Supplementary Table S3) were then used in generalized linear mixed-effect models as predictors for each outcome, adjusting for covariates, including MMT (categorized as “no MMT”, “1–2 MMT”, “ > 2 MMT”).

To adjust for multiple testing (56 regressions in total: crude and adjusted models for three outcomes and five exposures; the latent class analyses for each outcome, and the stratified analyses), the statistical significance level was set at α = 0.001, using the Sidak method^47^. Non-standardized regression coefficients (β) and 99.9% confidence intervals (99.9%CI) were estimated and then combined for the multiple imputed datasets. All analyses were performed with the statistical software SAS version 9.4 (Statistical Analyses System, SAS Institute Inc., Cary, NC, USA).

### Sensitivity analyses

In post-hoc analyses, the associations in step two were stratified by sex, family structure (one- vs. two-parent) and parental education attainment, adjusted for remaining covariates, in order to explore underlying differences. To account for self- and proxy-reported data by adolescents and parents, we stratified the impulsivity analysis group by age group (< 12 years vs. ≥ 12 years) and further adjusted for continuous age.

#### Informed consent

All procedures followed were in accordance with the ethical standards of the Helsinki Declaration of 1975, and its later amendments. Ethical approval was obtained from local institutional review boards at each study centre. Informed consent was obtained from all participants included in the study.

## Results

Characteristics of the analysis groups are provided in Table 1 and Supplementary Table S4. Information on emotion-driven impulsiveness was provided by 3261 children aged 9.9–17.9 years (mean/SD = 13.6/1.1). The impulsivity score ranged from 12 to 48 (mean/SD = 25.1/7.6). Data on decision-making ability was provided by 4046 children aged 8–17.9 years (mean/SD = 11.6/1.9). The DMA score ranged from − 100 to + 100 (median =  − 6.0, IQR = -18/0). Information on CIF was provided by 3441 children aged 8–17.9 years (mean/SD = 11.7/2.0). The CIF score ranged from 0 to 39 (median = 11.0, IQR = 7/15). Across all children, about half of them were females and 26% of them had overweight/obesity. Additionally, half of children had parents with high educational background. Characteristics of children not completing the cognitive tests are depicted in Supplementary Table S5.

**Table 1:** Characteristics of European children and adolescents who took part in the cognitive tests for assessing impulsivity, cognitive inflexibility and decision-making ability.

Covariables	Cognitive outcomes					
	Impulsivity		Decision-making ability		Cognitive inflexibility	
	N	%^a^	N	%	N	%
All	32,610	100.0	40,460	100.0	34,410	100.0
Sex						
Boys	15,500	47.5	20,280	50.1	17,230	50.1
Girls	17,110	52.5	20,180	49.9	17,180	49.9
Parental education attainment						
Low	1834	5.6	2492	6.2	2034	5.9
Medium	14,128	43.3	18,022	44.5	15,191	44.1
High	16,648	51.1	19,946	49.3	17,185	49.9
Weight status						
Underweight	2393	7.3	3043	7.5	2634	7.7
Normal weight	21,589	66.2	26,531	65.6	22,763	66.2
Overweight/obese	8628	26.5	10,886	26.9	9013	26.2
Pubertal status						
Pre-pubertal or early pubertal	7369	22.6	24,094	59.6	19,883	57.8
Pubertal	25,241	77.4	16,366	40.4	14,527	42.2
Being an only child						
Yes	7307	22.4	8438	20.9	7202	20.9
No	25,303	77.6	32,022	79.1	27,208	79.1
Family structure						
One-parent	4009	12.3	5331	13.2	4610	13.4
Two-parent	28,601	87.7	35,129	86.8	29,800	86.6
Country						
Italy	5630	17.3	8210	20.3	6300	18.3
Estonia	4900	15.0	9780	24.2	8760	25.5
Cyprus	7810	23.9	1110	2.7	870	2.5
Belgium	710	2.2	1560	3.9	1400	4.1
Poland^b^	760	2.3	–	–	–	–
Sweden	2420	7.4	3300	8.2	2400	7.0
Germany	4570	14.0	6470	16.0	5960	17.3
Hungary	4210	12.9	7850	19.4	6840	19.9
Spain	1600	4.9	2180	5.4	1880	5.5
Age, range (mean, SD)	9.9–17.9 (13.6, 1.1)		8.0–17.9 (11.6, 1.9)		8.0–17.9 (11.7, 2.0)	
Media rules, range (median, IQR)^c^	0–9 (6.0, 5/7)		0–9 (7.0, 5/7)		0–9 (7.0, 5/7)	
Impulsivity score, range (mean, SD)	12–48 (25.1, 7.6)					
Decision making score, range (median, IQR)^d^			− 100–+ 100 (− 6.0, − 18/0)			
Cognitive inflexibility score, range (median, IQR)					0–39 (11.0, 7/15)	

^a^ Results are based on imputed samples (10 replications). Due to rounding of decimals, percentages may not add up to 100%. ^b^ Polish children and adolescents reported information only on impulsivity, as the computerized tests were not performed in this sample. ^c^ Abbreviations: IQR- interquartile range. ^d^ For DMA and CIF, the median and IQR are reported because the scores were skewed. However, the values of skewness (0.8 and -0.14 respectively) did not exceed the threshold (-1, + 1), hence transformation was not necessary^75^.

### Digital media exposure in association with cognitive functioning

The results of the adjusted associations between individual DM use and measures of cognitive functioning are shown in Table 2**.** One additional hour of smartphone and internet exposure daily were associated with higher impulsivity score (β_smartphone_, 0.74; 99.9%CI 0.42–1.07; β_internet_, 0.57; 99.9%CI 0.28–0.85), after correcting for multiple testing. Smartphone exposure was positively associated with CIF (β, 0.32; 99.9%CI -0.02–0.66), although not statistically significant after correcting for multiple testing. Positive associations between MMT and impulsivity (β, 0.73; 99.9%CI 0.35–1.12) and CIF (β, 0.39; 99.9%CI 0.01–0.77) were also observed. Although not statistically significant, smartphone exposure (β, -0.47; 99.9%CI − 1.50–0.55) and MMT (β, − 0.70; 99.9%CI − 1.82–0.41) were inversely associated with DMA, while PC (β, 0.52; 99.9%CI − 0.72–1.77) and internet exposure were positively associated with DMA.

**Table 2:** The association of digital media exposure with impulsivity, cognitive inflexibility and decision-making ability in European children and adolescents.

	Digital media exposure ^a^									
	TV use		PC use		Smartphone use		Internet use		Media multitasking	
	Crude β (99.9%CI)	Adjusted β (99.9%CI)^b^	Crude β (99.9% CI)	Adjusted β (99.9%CI)^b^	Crude β (99.9% CI)	Adjusted β (99.9%CI)^b^	Crude β (99.9% CI)	Adjusted β (99.9%CI)^b^	Crude β (99.9% CI)	Adjusted β (99.9%CI)^b^
Impulsivity (N = 3260) ^c^	0.42 (− 0.03,0.86)	0.22 (− 0.20,0.64)	0.22 (− 0.22, 0.66)	0.33 (− 0.11, 0.77)	**1.00 (0.66, 1.33) **^**d**^	**0.74 (0.42, 1.07)**	**0.68 (0.39, 0.97)**	**0.57 (0.28, 0.85)**	**1.11 (0.71, 1.51)**	**0.73 (0.35, 1.12)**
Cognitive inflexibility (N = 3441)	− 0.08 (− 0.46, 0.28)	− 0.03 (− 0.40, 0.34)	− 0.24 (− 0.62, 0.14)	0.07 (− 0.33, 0.48)	0.11 (− 0.20, 0.43)	0.32 (− 0.02, 0.66)	− **0.35 (**− **0.60,** − **0.10)**	0.001 (− 0.28, 0.29)	0.20 (− 0.15, 0.55)	**0.39 (0.01, 0.77)**
Decision-making ability N = 4046)	− 0.32 (− 1.40, 0.76)	− 0.16 (− 1.28, 0.96)	− 0.15 (− 1.27, 0.96)	0.52 (− 0.72, 1.77)	− 0.83 (− 1.75, 0.09)	− 0.47 (− 1.50, 0.55)	− 0.37 (− 1.10, 0.36)	0.23 (− 0.66, 1.14)	− **1.05 (**− **2.06,** − **0.05)**	-0.70 (-1.82, 0.41)

^a^ Models are based on regressing single DM exposures on each of the outcomes. ^b^ All models are adjusted for basic confounders including sex, continuous age, parental education level (low, medium, high), country of residence, total sleep duration (continuous), pubertal status (pre- or early pubertal vs. pubertal), well-being score (continuous), in addition to media rules at home (continuous), being an only child (yes vs. no) and family structure (one-parent vs. two-parent family). In all models, a random effect for family-id was included, to consider family influences, thus to partially account for genetic factors. ^c^ Due to missing value for the family id, one participant was not included in the analysis. ^d^ Bold numbers indicate statistical significance based on 99.9% confidence intervals.

### Latent profiles of DM use in association with cognitive functioning

The LCA of four latent DM profiles showed the lowest BIC and a clear interpretable distinction of conditional probabilities for the respective variables (Supplementary Table S3). Profile names were chosen based on the highest conditional probabilities. The majority of participants (57%) had low usage of all media types, i.e., < 1 h/day for each media. This was considered the most favorable DM profile and was thus used as reference category in regression models of step two. Circa 13% of participants had high DM use except smartphone, while 10% had high smartphone and internet use, but medium TV and low PC use. Roughly 20% of participants had medium TV/internet, but low smartphone/PC use.

The adjusted regression models in step two showed that participants with “high DM use, except smartphone”, had an almost 2-point higher impulsivity score (β, 1.81; 99.9%CI 0.67–2.96) compared to those with low use of all media, independent of MMT (Table 3). This association remained significant among girls (β, 2.32; 99.9%CI 0.66–3.99), adolescents (β, 1.80; 99.9%CI 0.65–2.95) and those living in two-parent family (β, 1.79; 99.9%CI 0.54–3.04). The stratified analyses by parental education level (Supplementary Table S6) showed positive associations across all strata. Statistically significant associations were observed in participants from families with a medium educational background (β, 2.21; 99.9%CI 0.41–4.01). Children and adolescents with “high smartphone/internet, medium TV/low PC use” also showed higher impulsivity scores (β, 1.67, 99.9%CI 0.47–2.87), especially among girls (β, 1.73, 99.9%CI 0.21, 3.24), adolescents (β, 1.62, 99.9%CI 0.42, 2.82), and participants from two-parent families (β, 1.88, 99.9%CI 0.60, 3.17) (Table 3).

**Table 3:** Association of latent profiles of digital media use with impulsivity in European children and adolescents.

	Media use profiles ^a^ (Ref: Low DM use)			Media multitasking (Ref: No MMT)	
	High DM use, except smartphone	High smartphone/internet, medium TV/low PC	Medium TV/internet, low smartphone/PC	1–2 MMT	> 2 MMT
	Adjusted β (99.9% CI)	Adjusted β (99.9% CI)	Adjusted β (99.9% CI)	Adjusted β (99.9% CI)	Adjusted β (99.9% CI)
Analysis group (N = 3260) ^b^	**1.81 (0.67, 2.96) **^**c**^	**1.67 (0.47, 2.87)**	0.55 (− 0.55, 1.66)	0.97 (− 0.07, 2.02)	**1.61 (0.2, 3.03)**
Boys (N = 1549)	1.35 (− 0.26, 2.96)	1.80 (− 0.19, 3.80)	− 0.16 (− 1.66, 1.33)	0.74 (− 0.71, 2.20)	0.96 (− 1.01, 2.94)
Girls (N = 1711)	**2.32 (0.66, 3.99)**	**1.73 (0.21, 3.24)**	1.31 (− 0.33, 2.96)	1.21 (− 0.29, 2.71)	**2.30 (0.27, 4.34)**
Children (N = 38)	1.22 (− 9.57, 12.02)	− 4.19 (− 22.2, 13.9)	1.52 (− 7.97, 11.3)	1.04 (− 6.81, 8.91)	10.15 (− 6.75, 27.0)
Adolescents (N = 3222)	**1.80 (0.65, 2.95)**	**1.62 (0.42, 2.82)**	0.53 (− 0.58, 1.64)	1.00 (− 0.04, 2.06)	**1.62 (0.20, 3.04)**
One parent family (N = 397)	1.94 (− 1.34, 5.23)	− 0.04 (− 3.81, 3.71)	− 0.02 (− 3.18, 3.14)	1.34 (− 1.89, 4.58)	2.32 (− 1.98, 6.63)
Two parent family (N = 2863)	**1.79 (0.54, 3.04)**	**1.88 (0.60, 3.17)**	0.62 (− 0.58, 1.82)	0.91 (− 0.21, 2.05)	**1.54 (0.02, 3.05)**

^a^ Models are based on regressing the latent profiles of DM exposure on impulsivity, adjusting for basic confounders, including sex (not in the models stratified by sex), continuous age, parental education level (low, medium vs. high), country of residence, total sleep duration (continuous), pubertal status (pre- or early pubertal vs. pubertal), well-being score (continuous), in addition to media rules at home (continuous), being an only child (yes vs. no), family structure (one- vs. two-parent family; not in the models stratified by family structure) and media multitasking (no MMT, 1–2 MMT vs. > 2 MMT). In all models, a random effect for family id was included, to consider family influences and to partially account for genetic factors influencing the cognitive functioning. ^b^ Due to missing value for the family id, one participant was not included in the analyses. ^c^ Bold numbers indicate statistical significance based on 99.9% confidence intervals.

Table 4 shows the association between latent profiles of DM use and cognitive inflexibility in children and adolescents. Although not statistically significant, the results indicate a negative association between the profile “high DM use, except smartphone” and CIF. In contrast, a positive association between “high smartphone/internet, medium TV/low PC use” profile and CIF was observed in the overall sample and across strata, except for boys (Table 4) and for participants from families with low educational level (Supplementary Table S7).

**Table 4:** Association of latent profiles of digital media use with cognitive inflexibility in European children and adolescents.

	Media use profiles ^a^ (Ref: Low DM use)			Media multitasking (Ref: No MMT)	
	High DM use, except smartphone	High smartphone/internet, medium TV/low PC	Medium TV/internet, low smartphone/PC	1–2 MMT	> 2 MMT
	Adjusted β (99.9% CI)	Adjusted β (99.9% CI)	Adjusted β (99.9% CI)	Adjusted β (99.9% CI)	Adjusted β (99.9% CI)
Analysis group (N = 3441)	− 0.37 (− 1.49, 0.75)	0.47 (− 0.79, 1.74)	− 0.06 (− 0.97, 0.84)	0.45 (− 0.34, 1.24)	1.13 (− 0.29, 2.56)
Boys (N = 1723)	− 0.62 (− 2.09, 0.85)	− 0.12 (− 2.19, 1.84)	− 0.21 (− 1.39, 0.96)	0.66 (− 0.37, 1.70)	1.23 (− 0.68, 3.16)
Girls (N = 1718)	− 0.15 (− 1.94, 1.63)	0.95 (− 0.81, 2.72)	0.14 (− 1.33, 1.62)	0.23 (− 0.92, 1.40)	0.97 (− 1.13, 3.08)
One-parent family (N = 459)	− 0.53 (− 3.86, 2.78)	0.56 (− 3.04, 4.16)	− 0.004 (− 3.16, 3.15)	0.40 (− 1.95, 2.75)	1.07 (− 2.88, 5.03)
Two-parent family (N = 2982)	− 0.34 (− 1.56, 0.88)	0.49 (− 0.90, 1.88)	− 0.08 (− 1.08, 0.90)	0.45 (− 0.38, 1.29)	1.18 (− 0.37, 2.74)

^a^ Models are based on regressing the latent profiles of DM exposure on cognitive inflexibility on the same model, adjusting for basic confounders, including sex (not in the models stratified by sex), continuous age, parental education level (low, medium vs. high), country of residence, total sleep duration (hours/day), pubertal status (pre- or early pubertal vs. pubertal status), well-being score (continuous), in addition to media rules at home (continuous), being an only child (yes vs. no), family structure (one- vs. two parent family; not in the models stratified by family structure) and media multitasking (no MMT, 1–2 MMT, > 2 MMT). In all models, a random effect for family id was included, to consider family influences and to partially account for genetic factors influencing the cognitive functioning. None of the associations were statistically significant after adjustment for multiple testing.

The adjusted associations between latent DM profiles and DMA, depicted in Table 5 and Supplementary Table S8, showed a positive, but not statistically significant association for the profile of “high DM use, except smartphone” and DMA. Children with high smartphone/internet, but medium TV/low PC use” showed lower DMA scores across all strata. Although not statistically significant, participants living in one-parent households (Table 5) showed more than 4-point lower DMA score when exposed to smartphones/internet for > 2 h/day (β, − 4.36; 99.9%CI, -16.1–7.43) compared to low use of all media (< 1 h/day).

**Table 5:** Association of latent profiles of digital media use with decision-making ability in European children and adolescents.

	Media use profiles ^a^ (Ref: Low DM use)			Media multitasking (Ref: No MMT)	
	High DM use, except smartphone	High smartphone/internet, medium TV/low PC	Medium TV/internet, low smartphone/PC	1–2 MMT	> 2 MMT
	Adjusted β (99.9% CI)	Adjusted β (99.9% CI)	Adjusted β (99.9% CI)	Adjusted β (99.9% CI)	Adjusted β (99.9% CI)
Analysis group (N = 4046)	1.32 (− 2.14, 4.79)	− 1.40 (− 5.12, 2.32)	− 0.82 (− 3.54, 1.90)	− 1.24 (− 3.50, 1.00)	− 1.46 (− 5.80, 2.86)
Boys (N = 2028)	1.71 (− 3.17, 6.6)	− 3.05 (− 9.73, 3.62)	− 0.78 (− 4.59, 3.03)	− 2.02 (− 5.37, 1.33)	− 1.58 (− 8.1, 4.94)
Girls (N = 2018)	− 0.16 (− 5.02, 4.7)	− 0.82 (− 5.44, 3.79)	− 1.17 (− 5.13, 2.78)	− 0.22 (− 3.25, 2.80)	− 1.07 (− 6.72, 4.58)
One-parent family (N = 528)	− 0.10 (− 11.0, 10.8)	− 4.37 (− 16.1, 7.43)	− 0.46 (− 8.72, 7.79)	− 0.64 (− 7.73, 6.45)	− 1.84 (− 13.8, 10.1)
Two-parent family (N = 3518)	1.54 (− 2.37, 5.56)	− 0.89 (− 5.0, 3.21)	− 1.03 (− 4.0, 1.93)	− 1.25 (− 3.65, 1.13)	− 1.24 (− 5.95, 3.47)

^a^ Models are based on regressing the latent profiles of DM exposure on the decision-making ability on the same model, adjusting for covariates including sex (not in the models stratified by sex), continuous age, parental education level (low, medium vs. high), country of residence, total sleep duration (hours/day), pubertal status (pre- or early pubertal vs. pubertal status), well-being score (continuous), in addition to media rules at home (continuous), being an only child (yes vs. no), family structure (one- vs. two parent family; not in the models stratified by family structure) and media multitasking (no MMT, 1–2 MMT, > 2 MMT). In all models, a random effect for family id was included, to consider family influences and to partially account for genetic factors influencing the cognitive function. None of the associations were statistically significant after adjustment for multiple testing.

## Discussion

This cross-sectional study using data from European children and adolescents shows that the duration of exposure to contemporary DM, including smartphones and internet as well as media multitasking, are positively associated with emotion-driven impulsiveness and cognitive inflexibility, independent of well-being, sleep duration and weight status. The strength of these associations differed by sex and family structure.

Longer exposure to smartphones, internet and MMT was associated with a higher impulsivity score among children and adolescents in the present study, while no association was observed for non-internet-based media including TV and PC use. These findings suggest that the perpetual flow of information and input received simultaneously from the online environment such as emails, notifications and SM posts, may act as stressors. It is hypothesized that these exposures exceed the cognitive capacity of youth to handle and process that information, thus leading to “digital stress”^48^. Children and adolescents may be particularly vulnerable to digital stress because the neuronal myelination and synaptic pruning within the parietal and prefrontal cortex (areas related to attention control and delayed reinforcement) are not fully developed, leading to compromised emotional-regulation^49^ and reduced control of impulses^50^. These findings are worrisome considering the obesogenic (digital) food environment. The impact of smartphones and internet on EDI might lie in the pathway of mindless eating in front of screens, especially in reward-seeking contexts. Moreover, the prolific content accessible via internet-based DM (smartphones, SM platforms) which provides short and continuous gratifications that may activate the reward system (caudate, insula) and subsequent emotional and behavioural responses, such as snacking^24^. Another potential explanation may lie in the fact that DM displaces (real-life) social interactions such as parent–child, sibling- or peer relationships, often known as technoference. Social interactions are crucial for a healthy development because they built the foundation of processes related with personality and cognitive development, such as emotion regulation^51^. The interference of DM with parent–child interactions may compete with children’s ability to concentrate and regulate their emotions, leading to internalizing and externalizing problems like reduced ability to control impulses^52^.

Children and adolescents who used all DM except smartphone for > 2 h/day, had almost 2-point higher impulsivity score compared to children with low use of all media. Although the sole exposure to TV and PC was not associated with impulsivity, using them for prolonged duration in addition to constantly checking internet content seems to have a higher negative impact on children’s capabilities to regulate emotions. The association between “high smartphone/internet use, medium TV/low PC” and impulsivity was stronger among girls compared to boys, potentially because girls use smartphones and internet mainly for socializing and navigating SM, while boys mainly use them to play games^2^. Previous evidence shows that SM exposure impacts girls’ and adolescents’ psycho-emotional well-being^53^ and body-image^54^ via social comparisons over images posted on these platforms. This may lead to emotional overeating or restrained eating, as maladaptive coping strategies for relieving negative emotions^55^. Remarkably, neuro-developmental differences between children and adolescents might explain the stronger association between DM exposure and impulsivity in adolescents. The limbic subcortical system (affective/hot system) matures early on and the control system (cold) matures later in adolescence^56^, hence adolescents are more prone to engage in risky habits, also under digital stress.

Our results show that smartphone exposure and MMT were associated with higher cognitive inflexibility, suggesting that the digital environment may adversely impact youth’s ability to smoothly shift between tasks. Smartphones and MMT encourage high levels of flicking between information sources at the expense of brain circuitry needed to sustain concentration^57^. This may explain why smartphone use and MMT lead to poor academic performance in youth^27,28^. Additionally, repeated exposure to fast-paced content, like short-edited video segments in SM (e.g., Instagram reels) or online game applications might trigger individuals into seeking higher arousal levels, which in turn hamper engagement in activities that require sustained attention (e.g., homework)^58^. The frequency of checking smartphones and internet might also lie in the pathway of the aforementioned associations. One longitudinal study conducted among Japanese children of a similar age range observed that increasing internet use frequency was associated with reduced increases of the grey and white brain matter volume, which are responsible for attention control and executive functioning^16^. Although not significant, the negative association between prolonged exposure to all DM, except smartphone, and CIF suggests that children may be using those media for educational purposes and this could positively influence their mental multitasking abilities. Prolonged exposure to smartphones/internet but medium TV/low PC use was associated with higher CIF, indicating that smartphones particularly may disrupt children’s cognitive multitasking compared to other DM, as they are mostly used for entertainment purposes^59,60^, rather than for education^12^.

The adjusted associations between DM exposure and decision-making ability in a reward-related context were not statistically significant, but suggested a negative association of smartphone exposure and MMT with DMA. This aligns with a previous study where excessive smartphone use was related to reduced connectivity in the orbitofrontal cortex, a brain region related to DMA in reward-seeking behaviours^61^. One potential explanation could be that DM may interfere with children’s capacities to weigh short-term rewards against long-term negative outcomes, especially in a highly rewarding (digital) food environment (e.g., by consuming energy-dense foods). This is supported by previous research, which showed that children exposed to multiple DM tend to make unhealthy food choices^62^. Nevertheless, to our best knowledge, no other study has investigated the role of DM exposure on decision-making ability using the Hungry Donkey Test. Given the limited research in this area, our non-significant results should be interpreted with caution. Studies conducted in children of a similar age range using both the Hungry Donkey Test^63^ and Iowa Gambling Test^64^ have shown that DMA varies with age in a U-shaped curve. Younger children perform better in the task compared to early-adolescents, with performance becoming again better in late adolescents. This indicates that although DM use increases with age^46,65^, other mechanisms impact the development of DMA. The ventromedial prefrontal cortex, for instance, which is specialized in decision-making, functionally matures during adolescence and continues until young adulthood^66^. In our study, we measured the self-reported DM use duration, while previous studies that found significant association between DM exposure and activation of decision-making related brain areas, used the Smartphone^61^ and Internet Addiction Scale^67,68^. Thus, more longitudinal research is warranted to understand the extent and the underlying mechanisms (e.g., structural changes in the brain) via which DM exposure may impact DMA, using more detailed measures of DM, including SM exposure.

### Strengths and limitations

This is the first observational study to investigate the role of DM exposure on several measures of cognition in European children and adolescents. In contrast to most other studies focusing only on TV and video-gaming, we examined various DM exposures including contemporary DM like smartphone and internet, as well as MMT. Although all the single media (TV, PC, internet and smartphones) are digital media, we aimed at examining the association of each media with measures of cognitive functioning, because the patterns of use and the content children and adolescents are exposed to differs from one media to another. Using LCA to identify underlying patterns of DM exposure represents an advantage. We accounted for various important confounders of the associations between DM use and cognition, including sleep, well-being, pubertal and weight status, and family structure. All analyses were corrected for multiple testing, strengthening the reliability of the reported associations.

The cross-sectional nature of our study limits our ability to draw causal conclusions. Hence, we cannot exclude that certain psychological characteristics and personality traits predispose certain forms of DM use. Applying the reverse causality hypothesis, it can be assumed that prior delays in cognitive functioning may have led to a prolonged DM use among children and adolescents. Recent evidence also suggests that genetic variants and neuro-biological mechanisms commonly observed in behavioural addictions (i.e., dopamine release) are related to the excessive use of smartphones, internet, and video-games^69–71^. On the other side, an increasing number of studies have suggested various potential mechanisms through which DM may lead to poor cognitive functioning in children (as already discussed in this paper: digital stress, technoference, overstimulation, reduced attention control or impact on brain structures). Given that research on this topic is still at its infancy, future longitudinal studies are warranted to investigate how DM exposure over time impacts cognitive functioning while accounting for genetic and psychological characteristics, including the cognitive abilities in early childhood. Of note, when we controlled for factors like psychosocial well-being and partially accounted for family influences, the observed associations between DM exposure and cognitive functioning remained robust. When comparing the characteristics of participants completing the cognitive tests vs. those who did not, we observed no differences (expect for age and subsequently for pubertal status). Hence, it is unlikely that results are affected by selection bias, although the external validity may be limited given the non-representative sampling frame for each included country. Recall bias may have led to an underreported EDI, as the impulsivity sub-scale was self-reported. DM exposure was measured based on self-reports, thus a recall and social-desirability bias may have resulted to over- and under-estimation of DM use^72^. However, previous studies have shown that self-reported DM usage (e.g., smartphones) adequately distinguished between high and low use among adolescents^73^. The recency of the data is a limitation, as the digital environment and media skills of children have dramatically changed since 2013/2014. Our assessment of smartphone duration included the exposure to content such as TV shows, music videos or movies. Smartphones can be used for various purposes both offline and online, including playing games (offline) or social interactions (e.g., video-calling, texting etc.), which we could not account for with the available data. MMT was defined as the simultaneous use of a computer with other media, without considering smartphone and SM use, which are also significant contributors to MMT behaviour^74^. Therefore, the observed associations between MMT and cognitive functioning could be much more prominent in real life. We urge future studies to consider all sources of screen time and MMT to capture the complete picture of DM exposure during childhood. Moreover, we did not distinguish between smartphone and internet use for academic and entertainment purposes, which might lead to different results, and future studies should examine this hypothesis. We also lacked information on ease of access to DM at home, although we partly accounted for this by using information on media rules at home and MMT. Finally, we could not obtain information on SM exposure (Instagram, TikTok)^60^ and we urge further research to investigate the role of SM on children’s cognitive functioning, by considering the patterns of use (duration or problematic/addictive use of SM) and type of content provided.

## Conclusions

Smartphone, internet and media multitasking were found to be positively associated with emotion-driven impulsiveness and cognitive inflexibility, independent of psychosocial well-being and family structure. Our study provides evidence on a potential underlying mechanism by which DM exposure affects cognitive development and related health behaviours. These findings ask for parents, paediatricians and policy makers to help youth implement sound media use habits, in order to build the foundation for developing healthy psycho-physiological resilience against the likely adverse impact of digital environment.

### Supplementary Information

Supplementary Information.

## Data Availability

Due to the sensitive nature of data collected, ethical restrictions prohibit the authors from making the minimal data set publicly available. Each cohort centre received approval of the corresponding local Ethical Commission and participants did not provide consent for data sharing. Data are available on request and all requests need approval by the Steering Committee of the study. Interested researchers can contact the study co-ordinator (ahrens@leibniz-bips.de) to request data access. All requests for accessing data of the I.Family cohort are discussed on a case-by-case basis by the Steering Committee.
