# Author Correction: Digital media exposure and cognitive functioning in European children and adolescents of the I.Family study

**DOI:** 10.1038/s41598-023-49411-8

**Published:** 2023-12-18

**Authors:** Elida Sina, Christoph Buck, Wolfgang Ahrens, Juul M. J. Coumans, Gabriele Eiben, Annarita Formisano, Lauren Lissner, Artur Mazur, Nathalie Michels, Dénes Molnar, Luis A. Moreno, Valeria Pala, Hermann Pohlabeln, Lucia Reisch, Michael Tornaritis, Toomas Veidebaum, Antje Hebestreit, I. Family consortium

**Affiliations:** 1 Leibniz Institute for Prevention Research and Epidemiology - BIPS, Bremen, Germany; 2 Faculty of Mathematics and Computer Science, Institute of Statistics, University of Bremen, Bremen, Germany; 3 Teaching and Learning Centre, Open University of the Netherlands, Heerlen, The Netherlands; 4 Department of Public Health, School of Health Sciences, University of Skövde, Skövde, Sweden; 5 Institute of Food Sciences, National Research Council, Avellino, Italy; 6 School of Public Health and Community Medicine, Institute of Medicine, Sahlgrenska Academy, University of Gothenburg, Gothenburg, Sweden; 7 Faculty of Medicine, University of Rzeszów, Rzeszów, Poland; 8 Department of Public Health and Primary Care, Ghent University, Ghent, Belgium; 9 Department of Pediatrics, Medical School, University of Pécs, Pécs, Hungary; 10 GENUD (Growth, Exercise, Nutrition and Development) Research Group, Centro de Investigación Biomédica en Red Fisiopatología de La Obesidad y Nutrición (CIBERObn), Instituto Agroalimentario de Aragón (IA2), Instituto de Investigación Sanitaria Aragón (IIS Aragón), University of Zaragoza, Zaragoza, Spain; 11 Department of Epidemiology and Data Science, Fondazione IRCCS, Istituto Nazionale Dei Tumori, Milan, Italy; 12 Cambridge Judge Business School, University of Cambridge, Cambridge, UK; 13 Research and Education Institute of Child Health, Strovolos, Cyprus; 14 Department of Chronic Diseases, National Institute for Health Development, Tallinn, Estonia

Correction to: *Scientific Reports* 10.1038/s41598-023-45944-0, published online 01 November 2023

The original version of this Article contained an error in the I. Family consortium, where authors Christoph Buck, Wolfgang Ahrens, Juul M. J. Coumans, Luis A. Moreno, Hermann Pohlabeln, Michael Tornaritis, Toomas Veidebaum and Antje Hebestreit were omitted as Consortium Members. The correct list of authors and their affiliations appears below:

**I. Family consortium**

Elida Sina^1^, Christoph Buck^1^, Wolfgang Ahrens^2^, Juul M. J. Coumans^3^, Gabriele Eiben^4^, Annarita Formisano^5^, Lauren Lissner^6^, Artur Mazur^7^, Nathalie Michels^8^, Dénes Molnar^9^, Luis A. Moreno^10^, Valeria Pala^11^, Hermann Pohlabeln^1^, Lucia Reisch^1,12^, Michael Tornaritis^13^, Toomas Veidebaum^14^, Antje Hebestreit^1^

1 Leibniz Institute for Prevention Research and Epidemiology - BIPS, Bremen, Germany.

2 Faculty of Mathematics and Computer Science, Institute of Statistics, University of Bremen, Bremen, Germany.

3 Teaching and Learning Centre, Open University of the Netherlands, Heerlen, The Netherlands.

4 Department of Public Health, School of Health Sciences, University of Skövde, Skövde, Sweden.

5 Institute of Food Sciences, National Research Council, Avellino, Italy.

6 School of Public Health and Community Medicine, Institute of Medicine, Sahlgrenska Academy, University of Gothenburg, Gothenburg, Sweden.

7 Faculty of Medicine, University of Rzeszów, Rzeszów, Poland.

8 Department of Public Health and Primary Care, Ghent University, Ghent, Belgium.

9 Department of Pediatrics, Medical School, University of Pécs, Pécs, Hungary.

10 GENUD (Growth, Exercise, Nutrition and Development) Research Group, Centro de Investigación Biomédica en Red Fisiopatología de La Obesidad y Nutrición (CIBERObn), Instituto Agroalimentario de Aragón (IA2), Instituto de Investigación Sanitaria Aragón (IIS Aragón), University of Zaragoza, Zaragoza, Spain.

11 Department of Epidemiology and Data Science, Fondazione IRCCS, Istituto Nazionale Dei Tumori, Milan, Italy.

12 Cambridge Judge Business School, University of Cambridge, Cambridge, UK.

13 Research and Education Institute of Child Health, Strovolos, Cyprus.

14 Department of Chronic Diseases, National Institute for Health Development, Tallinn, Estonia.

The original Article has been corrected.

