# Supplementary material for: Digital media exposure and cognitive functioning in European children and adolescents of the I.Family study
Source: Sci Rep. 2023 Nov 1;13:18855. doi: 10.1038/s41598-023-45944-0 (PMC10620404; doi:10.1038/s41598-023-45944-0)
Supplement: Supplementary file 1 — Supplementary Information. [file 41598_2023_45944_MOESM1_ESM.docx]

**Digital media exposure and cognition functioning in European children and adolescents of the I.Family study**

**Supplementary Figure S1:** Flow chart depicting the number of participants from I.Family study included in the current analyses

**Supplementary Methods**

**Supplementary Table S1.** Characteristics of children and adolescents in the non-imputed and the imputed sample

**Supplementary Table S2.** The selection of model fit of the latent profiles of digital media use

**Supplementary Table S3.** Latent profiles of digital media (DM) use and highest conditional probabilities (ρ-estimate) of categories within each profile

**Supplementary Table S4**. Digital media exposure (in categories) by cognitive outcome

**Supplementary Table S5.** Characteristics of children and adolescents not completing the cognitive tests

**Supplementary Table S6.** The association of latent profiles of digital media exposure with impulsivity in European children and adolescents, by parental education attainment

**Supplementary Table S7.** The association of latent profiles of digital media exposure with cognitive inflexibility in European children and adolescents, by parental education attainment

**Supplementary Table S8.** The association of latent profiles of digital media exposure with decision-making ability in European children and adolescents, by parental education attainment

**Supplementary References**

- 8,578 parents were excluded
- 6 duplicates

10676 children and adolescents

19,258 participants (children, adolescents and parents) were included in the I. Family study

8673 children and adolescents after exclusion criteria were met

Cognitive Inflexibility: 3441 children and adolescents

2003 participants excluded:

- 106 participants with TV/PC >50h/week
- 43 participants with TV/PC=0 h/week
- 1742 participants <8 years
- 112 participants with ADHD
- 4621 participants did not complete the test
- 6 completed <100 trials
- 4639 participants did not complete the test
- 520 completed <1 category
- 7 participants <64 trials
- 66 were color blind
- 5273 participants did not complete the scale
- 139 participants with incomplete items

Decision-making ability: 4046 children and adolescents

Impulsivity: 3261 children and adolescents

**Supplementary Figure S1.** Flow chart depicting the number of participants from I.Family study included in the current analyses

**Supplementary Methods**

**Exclusion Criteria**

Children with implausible screen-time (TV/PC >50 h/week, N=106; and TV/PC=0 h/week, N=43) were excluded. To account for internet and smartphone dependency, we did not set boundaries on internet and smartphone use. Children who were diagnosed with attention-deficit hyperactivity disorder (ADHD) by a physician (N=112), as reported by parents using the Health and Medical History Questionnaire, were also excluded. After the primary exclusion criteria were met (N=8673), further secondary exclusion criteria were applied (invalid data on the cognitive tests or incomplete items for the impulsivity questionnaire, eFigure 1), hence the size of the analysis group varied across the cognitive tests.

**Measurement of digital media (DM) use**

Participants reported the duration of DM use for weekdays and weekend days separately, including TV/DVD/video, computer/game console (PC), and internet use as: not at all, <30 min/day, 30 min-1 h/day, about 1–2 h/day, about 2–3 h/day and >3 h/day”^1^. Internet users could also select the option of “I’m online more or less all day/night”. To obtain information on offline PC use and to prevent a potential overlap with internet use, we explicitly asked “How long do you usually sit at a computer/game console per day? (Please disregard the time spent on internet)”.

**Potential confounders**

Parents self-reported their highest education attainment according to the International Standard Classification of Education ^2^, classified as “low”, “medium” or “high”. As puberty influences physiological (e.g., hormonal changes), psychosocial and behavioural processes^3^, children aged ≥ 8 years provided information about the breaking of the voice for boys and onset of menarche for girls^4^. Pubertal status was then classified as pre-pubertal/early pubertal, and pubertal status. Information on maturation in Tanner stages (5 stages) based on the development of pubic hair (boys) and development of the breast (girls) was obtained and categorized into three categories: non-pubertal, peri-pubertal and pubertal ^5^. Parents completed a 9-item questionnaire on family rules about TV, video-games, smartphone use etc. Item examples include: “Do you have any rules about: i) -what your child/children is/are allowed to watch on TV; ii) -if your child/your children is/are allowed to have a profile on a social networking site like Facebook and how much time it/they can spend on it?”. Based on dichotomized answers (“yes” vs. “no”), a sum score on family media rules was calculated, ranging from 0 to 9. Psychosocial well-being was measured using the (16-item) four subscales of the KINDL^R^ Questionnaire for Measuring Health-Related Quality of Life (HRQoL) in children and adolescents: emotional well-being, self-esteem, family life, and relations to friends^6^. Initially, the questionnaire was developed in German but was then translated to English and other languages. Each survey centre used available language versions. A well-being score was calculated by summing up the 16 items scored on a five-point Likert scale ranging from “0” (Never) to “4” (All the time). Six items of the original scale were inversely coded to allow all items to run in the same direction. Consequently, the well-being score ranged from 0 to 48, with a higher score indicating higher psychosocial well‐being. The validity and reliability of the KINDL^R^ have been previously reported ^6^. Self-reported total daily sleep duration (hours/day) was calculated as sum of duration reported for nocturnal sleep and day-time napping at weekends and weekdays.

Weight and height of each child was measured in light clothing and in an overnight fasting state. Height was measured using a portable stadiometer (Seca GmbH & Co. KG., Hamburg, Germany) to the nearest 0.1 cm, and weight was measured using a Tanita scale (TANITA Europe GmbH, Sindelfingen, Germany) to the nearest 0.1 kg. We calculated body mass index (BMI) as weight divided by squared height, which was then transformed into age- and sex-specific z-scores for all children and adolescents. Participant’s weight status was then categorized as underweight, normal weight vs. overweight/obese according to the cut-offs of Cole et al^7^.

Parents provided information on family structure via a kinship and household interview^8^. Based on the number of children (<18 years) and adults (≥ 18 years) in the household, we calculated whether the participating child was an only child in that household. This information was used to control for the confounding effect of being an only child on cognitive development. Only children tend to pursuit more solitary activities, including solitary play with media and are deprived from sibling interactions. The latter facilitates emotional regulation and learning opportunities that enhance psychosocial skills and sociability^9,10^. Furthermore, the relation to the participating child for each household member was reported and codes were assigned for the relationship status that corresponded to ‘biological mother’, ‘biological father’, ‘biologically unrelated female adult’, ‘biologically unrelated male adult’, ‘any other adult’, ‘biological sibling’, ‘half-sibling’ or ‘non-biological sibling’. We assessed the number of parents in the household (either biologic or non-biologic parent) and then derived whether the participating child/adolescent lived in one-parent vs. two-parent family. Family structure plays a major role in children’s cognitive development, with those living in single-parent families having poorer academic achievement, lower emotional well-being^11^ and higher media use^12^.

**Supplementary Table S1**. **Characteristics of children and adolescents in the non-imputed and the imputed sample**

|  | **Non-Imputed Sample** | | **Complete Case Sample** | | **Imputed Sample^a^** | |
| --- | --- | --- | --- | --- | --- | --- |
| **Characteristics** | **N** | **%** | **N** | **%** | **N** | **%** |
| **All** | 8673 | 100.0 |  |  | 86730 | 100.0 |
| **Sex** |  |  |  |  |  |  |
| Boys | 4338 | 50.0 | 4338 | 50.0 | 43380 | 50.0 |
| Girls | 4335 | 50.0 | 4335 | 50.0 | 43350 | 50.0 |
| **Parental education status** |  |  |  |  |  |  |
| Missing | 1023 | 11.8 |  |  |  |  |
| Low | 406 | 4.7 | 406 | 5.3 | 5092 | 5.9 |
| Medium | 3325 | 38.3 | 3325 | 43.4 | 37386 | 43.1 |
| High | 3919 | 45.2 | 3919 | 51.2 | 44252 | 51.0 |
| **Puberty** |  |  |  |  |  |  |
| Missing | 1001 | 11.5 |  |  |  |  |
| Pre-pubertal or early pubertal | 4475 | 51.6 | 4475 | 58.3 | 50684 | 58.4 |
| Pubertal | 3197 | 36.9 | 3197 | 41.6 | 36046 | 41.6 |
| **Puberty in Tanner stages** |  |  |  |  |  |  |
| Missing | 2306 | 26.6 |  |  |  |  |
| Prepubertal | 1350 | 15.6 | 1350 | 21.2 | 18158 | 20.9 |
| Peripubertal | 3360 | 38.7 | 3360 | 52.7 | 45661 | 52.6 |
| Pubertal | 1657 | 19.1 | 1657 | 26.02 | 22911 | 26.4 |
| **Weight status** |  |  |  |  |  |  |
| Missing | 45 | 0.5 |  |  |  |  |
| Thinnes grade 3 | 24 | 0.3 | 24 | 0.2 | 248 | 0.3 |
| Thinnes grade 2 | 96 | 1.1 | 96 | 1.1 | 962 | 1.1 |
| Thinnes grade 1 | 584 | 6.7 | 584 | 6.7 | 5873 | 6.8 |
| Normal weight | 5587 | 64.4 | 5587 | 64.7 | 56177 | 64.8 |
| Overweight | 1662 | 19.2 | 1662 | 19.2 | 16688 | 19.2 |
| Obese | 675 | 7.8 | 675 | 7.8 | 6782 | 7.8 |
| **Family structure** |  |  |  |  |  |  |
| Missing | 1762 | 20.3 |  |  |  |  |
| One-parent | 865 | 10.0 | 865 | 12.5 | 10704 | 12.3 |
| Two-parent | 6046 | 69.7 | 6046 | 87.4 | 76026 | 87.6 |
| **Only child** |  |  |  |  |  |  |
| Missing | 1791 | 20.7 |  |  |  |  |
| Yes | 1298 | 15.0 | 1298 | 18.8 | 16109 | 18.6 |
| No | 5584 | 64.3 | 5584 | 81.1 | 70621 | 81.4 |
| **Country** |  |  |  |  |  |  |
| Italy | 1284 | 14.8 | 1284 | 14.8 | 12840 | 14.8 |
| Estonia | 1089 | 12.4 | 1089 | 12.4 | 10890 | 12.6 |
| Cyprus | 1924 | 22.2 | 1924 | 22.2 | 19240 | 22.2 |
| Belgium | 353 | 4.1 | 353 | 4.1 | 3530 | 4.1 |
| Poland | 693 | 8.0 | 693 | 8.0 | 6930 | 8.0 |
| Sweden | 732 | 8.4 | 732 | 8.4 | 7320 | 8.4 |
| Germany | 1102 | 12.7 | 1102 | 12.7 | 11020 | 12.7 |
| Hungary | 980 | 11.3 | 980 | 11.3 | 9800 | 11.3 |
| Spain | 516 | 5.9 | 516 | 5.9 | 5160 | 5.9 |
| Age range (mean, SD) | 8-17.9  (11.8, 2.1) | |  |  | 8-17.9  (11.8, 2.1) | |
| Sleep duration (mean, SD) | 4-18.1  (9.3, 1.0) | |  |  | 4-18.1  (9.3, 1.0) | |
| Psychosocial well-being score  (mean, SD) | 9-48  (39.2, 5.4) | |  |  | 9-48  (39.2, 5.4) | |

^a^ Results are based on imputed samples (10 replications).

As shown in **Supplementary Table S2**, the latent class analyses were conducted using two to six profiles of the individual media use, including TV, PC, internet and smartphone use in categories (<1 h/day; 1-2 h/day; >2 h/day). To select the model fit, diagnostic criteria were used, namely the Bayesian Information Criterion (BIC), the Akaike Information Criterion (AIC), degrees of freedom (DF, the lower, the better) and model convergence. Although the model with six and five latent profiles showed the lowest BIC, AIC and DF, these models did not converge, hence were not selected. Instead, the model with four latent DM profiles was chosen, as it showed the lowest BIC, AIC and DF compared to the models with three and two latent profiles.

**Supplementary Table S2.** The selection of model fit of the latent profiles of digital media use

| **Nr. of classes** | **Akaike Information Criterion**  **(AIC)** | **Bayesian Information Criterion (BIC)** | **Adjusted Bayesian Information Criterion (BIC)** | **Degrees of freedom (DF)** | **Entropy** | **Model converged** |
| --- | --- | --- | --- | --- | --- | --- |
| 2 | 6635 | 6794 | 6740 | 63 | 0.70 | Yes |
| 3 | 2215 | 2459 | 2376 | 54 | 0.60 | Yes |
| 4 | 1184 | 1512 | 1401 | 45 | 0.65 | Yes |
| 5 | 612 | 1024 | 884 | 36 | 0.66 | No |
| 6 | 465 | 962 | 793 | 27 | 0.66 | No |

**Supplementary Table S3.** Latent profiles of digital media (DM) use and highest conditional probabilities (ρ-estimate) of categories within each profile

| **Latent Profiles of DM exposure^a^** | |  | **Frequency^b^** | **%** |
| --- | --- | --- | --- | --- |
| **1. High DM use, except smartphone** | |  | 11374 | 13.11 |
| ***Variable*** | ***Category*** | ***ρ-estimate*** |  |  |
| TV use | >2 h/day | 0.5959 |  |  |
| PC use | >2 h/day | 0.4757 |  |  |
| Internet use | >2 h/day | 0.8498 |  |  |
| Smartphone use | <1 h/day | 0.5491 |  |  |
| **2. High smartphone/internet, medium TV/low PC** | |  | 8638 | 9.96 |
| ***Variable*** | ***Category*** | ***ρ-estimate*** |  |  |
| TV use | 1-2 h/day | 0.4360 |  |  |
| PC use | <1 h/day | 0.7539 |  |  |
| Internet use | >2 h/day | 0.4740 |  |  |
| Smartphone use | >2 h/day | 0.6589 |  |  |
| **3. Low DM use** | |  | 49212 | 56.74 |
| ***Variable*** | ***Category*** | ***ρ-estimate*** |  |  |
| TV use | <1 h/day | 0.5077 |  |  |
| PC use | <1 h/day | 0.9612 |  |  |
| Internet use | <1 h/day | 0.8913 |  |  |
| Smartphone use | <1 h/day | 0.9606 |  |  |
| **4. Medium TV/Internet, low smartphone/PC** |  |  |  |  |
| ***Variable*** | ***Category*** | ***ρ-estimate*** | 17506 | 20.18 |
| TV use | 1-2 h/day | 0.5197 |  |  |
| PC use | <1 h/day | 0.5348 |  |  |
| Internet use | 1-2 h/day | 0.4772 |  |  |
| Smartphone use | <1 h/day | 0.9067 |  |  |

^a^ Abbreviations: DM – digital media, IQR- interquartile range, TV- television viewing, PC – computer/game console use. ^b^ Results are based on imputed samples (10 replications).

**Supplementary Table S4** shows the duration of individual DM use among children and adolescents included in the analyses for the three cognitive outcomes. Among participants who provided information on emotion-driven impulsiveness, 30% of them had high TV exposure (>2 h/day), while 13.6% had high PC/game console use. With regard to more contemporary DM, 37% and 21% of participants had high internet and smartphone exposure, respectively. Additionally, more than 15% of participants engaged in more than two media multi-tasking activities simultaneously. Among children and adolescents who completed the test for assessing cognitive inflexibility, a quarter of them had high TV use, while circa 20% and 11% of them had high internet and smartphone exposure, respectively. Among children and adolescents who took part in the Hungry Donkey test, 20% and 10% of them had high use of internet and smartphones, respectively, while half of them engaged in at least one media multitasking activity.

**Supplementary Table S4. Digital media exposure of children and adolescents who took part in the cognitive tests**

| **Characteristics^a^** | **Impulsivity** | | **Decision-making ability** | | **Cognitive inflexibility** | |
| --- | --- | --- | --- | --- | --- | --- |
|  | **N** | **%**^b^ | **N** | **%** | **N** | **%** |
| All | 32610 | 100.0 | 40460 | 100.0 | 34410 | 100.0 |
| **Age group** |  |  |  |  |  |  |
| Children | 390 | 1.2 | 22950 | 56.7 | 14670 | 42.6 |
| Adolescents | 32220 | 98.8 | 17510 | 43.3 | 19740 | 57.4 |
| **TV use^c^** |  |  |  |  |  |  |
| Low | 10499 | 32.2 | 14833 | 36.7 | 12523 | 36.4 |
| Medium | 12262 | 37.6 | 15576 | 38.5 | 13264 | 38.5 |
| High | 9849 | 30.2 | 10051 | 24.8 | 8623 | 25.1 |
| **PC use** |  |  |  |  |  |  |
| Low | 21627 | 66.3 | 30608 | 75.7 | 25850 | 75.1 |
| Medium | 6561 | 20.1 | 6349 | 15.7 | 5420 | 15.8 |
| High | 4422 | 13.6 | 3503 | 8.7 | 3140 | 9.1 |
| **Internet use** |  |  |  |  |  |  |
| Low | 12346 | 37.9 | 24899 | 61.5 | 20678 | 60.1 |
| Medium | 8130 | 24.9 | 7301 | 18.0 | 6225 | 18.1 |
| High | 12134 | 37.2 | 8260 | 20.4 | 7507 | 21.8 |
| **Smartphone use** |  |  |  |  |  |  |
| Low | 21252 | 65.2 | 33140 | 81.9 | 27942 | 81.2 |
| Medium | 4476 | 13.7 | 3116 | 7.7 | 2657 | 7.7 |
| High | 6882 | 21.1 | 4204 | 10.4 | 3811 | 11.1 |
| **Media multi-tasking (MMT)** |  |  |  |  |  |  |
| No MMT | 6668 | 20.4 | 17309 | 42.8 | 14448 | 42.0 |
| 1-2 MMT | 20821 | 63.8 | 20253 | 50.1 | 17405 | 50.6 |
| >2 MMT | 5121 | 15.7 | 2898 | 7.2 | 2557 | 7.4 |

^a^ Results are based on imputed samples (10 replications). ^b^ Due to rounding of decimals, percentages may not add up to 100%. ^c^ Abbreviations: TV- television viewing, PC – computer/game console use, MMT – Media multitasking

**Supplementary Table S5** shows the characteristics of children and adolescents who did not take part in the cognitive tests, in comparison with children and adolescents who actually participated in the respective tests. The distribution of all variables was similar between excluded participants and those who were included for each test. We observed differences only with regard to age, given that the computerized test and the impulsivity questionnaire are developed for children aged ≥8 years, and thus were not administered to younger children. Subsequently, the distribution of the pubertal status is different for the excluded compared to the included sample, for each outcome.

**Supplementary Table S5. Characteristics of children and adolescents not completing the cognitive tests**

| **Characteristics**^a^ | **Impulsivity questionnaire not completed** | | **Impulsivity questionnaire completed** | | **Test for assessing decision-making not completed** | | **Test for assessing decision-making completed** | | **Test for assessing cognitive inflexibility not completed** | | **Test for assessing cognitive inflexibility completed** | |
| --- | --- | --- | --- | --- | --- | --- | --- | --- | --- | --- | --- | --- |
|  | **N** | **%^b^** | **N** | **%** | **N** | **%** | **N** | **%** | **N** | **%** | **N** | **%** |
| **All** | 54120 | 100.0 | 32610 | 100.0 | 46270 | 100.0 | 40460 | 100.0 | 52320 | 100.0 | 34410 | 100.0 |
| **Sex** |  |  |  |  |  |  |  |  |  |  |  |  |
| Boys | 27880 | 51.5 | 15500 | 47.5 | 23100 | 49.9 | 20280 | 50.1 | 26150 | 50.0 | 17230 | 50.1 |
| Girls | 26240 | 48.5 | 17110 | 52.5 | 23170 | 50.1 | 20180 | 49.9 | 26170 | 50.0 | 17180 | 49.9 |
| **Parental education attainment** |  |  |  |  |  |  |  |  |  |  |  |  |
| Low | 3258 | 6.0 | 1834 | 5.6 | 2600 | 5.6 | 2492 | 6.2 | 3058 | 5.8 | 2034 | 5.9 |
| Medium | 23258 | 43.0 | 14128 | 43.3 | 19364 | 41.9 | 18022 | 44.5 | 22195 | 42.4 | 15191 | 44.1 |
| High | 27604 | 51.0 | 16648 | 51.1 | 24306 | 52.5 | 19946 | 49.3 | 27067 | 51.7 | 17185 | 49.9 |
| **Pubertal status** |  |  |  |  |  |  |  |  |  |  |  |  |
| Pre-pubertal or early pubertal | 41146 | 76.0 | 7369 | 22.6 | 24421 | 52.8 | 24094 | 59.6 | 28632 | 54.7 | 19883 | 57.8 |
| Pubertal | 12974 | 24.0 | 25241 | 77.4 | 21849 | 47.2 | 16366 | 40.4 | 23688 | 45.3 | 14527 | 42.2 |
| **Weight status** |  |  |  |  |  |  |  |  |  |  |  |  |
| Underweight | 4690 | 8.7 | 2393 | 7.3 | 4040 | 8.7 | 3043 | 7.5 | 4449 | 8.5 | 2634 | 7.7 |
| Normal weight | 34588 | 63.9 | 21589 | 66.2 | 29646 | 64.1 | 26531 | 65.6 | 33414 | 63.9 | 22763 | 66.2 |
| Overweight/obese | 14842 | 27.4 | 8628 | 26.5 | 12584 | 27.2 | 10886 | 26.9 | 14457 | 27.6 | 9013 | 26.2 |
| **Family Structure** |  |  |  |  |  |  |  |  |  |  |  |  |
| One-parent | 6215 | 11.5 | 4009 | 12.3 | 4893 | 10.6 | 5331 | 13.2 | 5614 | 10.7 | 4610 | 13.4 |
| Two-parent | 47905 | 88.5 | 28601 | 87.7 | 41377 | 89.4 | 35129 | 86.8 | 46706 | 89.3 | 29800 | 86.6 |
| **Only child** |  |  |  |  |  |  |  |  |  |  |  |  |
| Yes | 8802 | 16.3 | 7307 | 22.4 | 7671 | 16.6 | 8438 | 20.9 | 8907 | 17.0 | 7202 | 20.9 |
| No | 45318 | 83.7 | 25303 | 77.6 | 38599 | 83.4 | 32022 | 79.1 | 43413 | 83.0 | 27208 | 79.1 |
| **Country** |  |  |  |  |  |  |  |  |  |  |  |  |
| Italy | 7210 | 13.3 | 5630 | 17.3 | 4630 | 10.0 | 8210 | 20.3 | 6540 | 12.5 | 6300 | 18.3 |
| Estonia | 5990 | 11.1 | 4900 | 15.0 | 1110 | 2.4 | 9780 | 24.2 | 2130 | 4.1 | 8760 | 25.5 |
| Cyprus | 11430 | 21.1 | 7810 | 23.9 | 18130 | 39.2 | 1110 | 2.7 | 18370 | 35.1 | 870 | 2.5 |
| Belgium | 2820 | 5.2 | 710 | 2.2 | 1970 | 4.3 | 1560 | 3.9 | 2130 | 4.1 | 1400 | 4.1 |
| Poland^c^ | 6170 | 11.4 | 760 | 2.3 | 6930 | 15.0 | - | - | 6930 | 13.2 | - | - |
| Sweden | 4900 | 9.1 | 2420 | 7.4 | 4020 | 8.7 | 3300 | 8.2 | 4920 | 9.4 | 2400 | 7.0 |
| Germany | 6450 | 11.9 | 4570 | 14.0 | 4550 | 9.8 | 6470 | 16.0 | 5060 | 9.7 | 5960 | 17.3 |
| Hungary | 5590 | 10.3 | 4210 | 12.9 | 1950 | 4.2 | 7850 | 19.4 | 2960 | 5.7 | 6840 | 19.9 |
| Spain | 3560 | 6.6 | 1600 | 4.9 | 2980 | 6.4 | 2180 | 5.4 | 3280 | 6.3 | 1880 | 5.5 |
| Age range  (mean, SD) | 8-17.9  (10.7, 1.7) | | 9.9 - 17.9  (13.6, 1.1) | | 8-17.9  (11.9, 2.2) | | 8.0 - 17.9  (11.6, 1.9) | | 8.0 - 17.9  (11.8, 2.2) | | 8.0 - 17.9  (11.7, 2.0) | |
| DM use (hours/day),  range (median, IQR)^d^ | 0.1-13.8  (2.6, 1.8/4) | | 0.1-13  (4.1, 2.6/6.2) | | 0.1-13.8  (3.2, 2.1/4.8) | | 0.1-13.00  (3.0, 1.9/4.7) | | 0.1-13.8  (3.1, 2.0/4.8) | | 0.1-12.9  (3.0, 1.9/4.9) | |
| Sleep duration (mean, SD) | 4.3-16  (9.4, 0.9) | | 4.0-18.1  (9.0, 1.2) | | 4-18.1  (9.3, 1.1) | | 5-16.8  (9.3, 1.0) | | 4-18.1  (9.3, 1.1) | | 5-16.3  (9.3, 1.0) | |
| Psychosocial well-being score  (mean, SD) | 9-48  (39.9, 5.1) | | 9-48  (38, 5.6) | | 9-48  (39.3, 5.3) | | 9-48  (39, 5.4) | | 9-48  (39.3, 5.3) | | 9-48  (39, 5.4) | |

^a^ Results are based on imputed samples (10 replications). ^b^ Due to rounding of decimals, percentages may not add up to 100%. ^c^ Polish children and adolescents provided information only on impulsivity, as the computerized tests were not performed in this sample. ^d^ Abbreviations: DM – digital media, IQR- interquartile range

The results of the adjusted regression models investigating the association between latent profiles of DM exposure and impulsivity, stratified by parental education attainment are shown in **Supplementary Table S6**. Participants with high DM use, except smartphone and who had parents with low educational background, showed almost a 2-point higher impulsivity score (β, 1.72, 99.9%CI, -3.58, 7.02) compared to children with low use of all media. Similarly, a positive and statistically significant association was observed between high DM use, except smartphone and impulsivity among children of parents with medium education attainment (β, 2.21, 99.9%CI, 0.41, 4.01). Moreover, children and adolescents with high smartphone/internet, medium TV/low PC use showed higher impulsivity score, independent of parental education.

**Supplementary Table S6. The association between latent profiles of digital media exposure with impulsivity in European children and adolescents, by parental education attainment**

|  | **Media use profiles ^a^**  **(**Ref: Low DM use**)** | | | **Media multi-tasking**  **(**Ref: No MMT**)** | |
| --- | --- | --- | --- | --- | --- |
|  | **High DM use, except smartphone** | **High smartphone/internet, medium TV/low PC** | **Medium TV/Internet, low smartphone/PC** | **1-2 MMT** | **>2 MMT** |
| **Parental education attainment** | **Adjusted β**  **(99.9% CI)** | **Adjusted β**  **(99.9% CI)** | **Adjusted β**  **(99.9% CI)** | **Adjusted β**  **(99.9% CI)** | **Adjusted β**  **(99.9% CI)** |
| **Low**  (N=179) | 1.72  (-3.58, 7.02) | 1.41  (-3.61, 6.43) | -0.02  (-5.69, 5.63) | 3.09  (-2.5, 8.68) | 3.60  (-2.71, 9.91) |
| **Medium**  (N=1408) ^b^ | **2.21^c^**  **(0.41, 4.01)** | **2.08**  **(0.19, 3.97)** | 0.16  (-1.67, 2.0) | 0.83  (-0.90, 2.56) | 1.38  (-0.91, 3.68) |
| **High**  (N=1673) | 1.54  (-0.06, 3.15) | 1.32  (-0.33, 2.97) | 0.91  (-0.54, 2.36) | 0.86  (-0.50, 2.54) | 1.66  (-0.23, 3.57) |

^a^ Models are based on regressing the latent profiles of DM exposure on impulsivity on the same model, adjusting for basic confounders, including sex, continuous age, country of residence, total sleep duration, pubertal status, well-being score, in addition to media rules at home, being an only child, family structure and media multi-tasking (in categories). In all models, a random effect for family id was included, to consider family influences and to partially account for genetic factors influencing the cognitive function. ^b^ Due to missing value for the family id, one participant was not included in the analysis. c Bold numbers indicate statistical significance based on 99.8% confidence intervals.

**Supplementary** **Table S7** depicts the associations between latent profiles of DM exposure and cognitive inflexibility among children and adolescents of parents with different educational attainment. Although not statistically significant after adjusting for multiple testing, the results show a negative association between the profile of high DM use except smartphone and cognitive inflexibility across all strata. Children of parents with medium and high education attainment who had a high smartphone/internet, but medium TV/low PC use showed higher cognitive inflexibility score, respectively, compared to children with low use of all media. A negative association between high smartphone/internet, but medium TV/low PC use and cognitive inflexibility was observed among children of parents with low educational attainment only (β, -0.39, 99.9%CI, -5.73, 4.94).

**Supplementary** **Table S7. The association of latent profiles of digital media exposure with cognitive inflexibility in European children and adolescents, by parental education attainment**

|  | **Media use profiles ^a^**  **(**Ref: Low DM use**)** | | | **Media multi-tasking**  **(**Ref: No MMT**)** | |
| --- | --- | --- | --- | --- | --- |
|  | **High DM use, except smartphone** | **High smartphone/internet, medium TV/low PC** | **Medium TV/Internet, low smartphone/PC** | **1-2 MMT** | **>2 MMT** |
| **Parental education attainment** | **Adjusted β**  **(99.9% CI)** | **Adjusted β**  **(99.9% CI)** | **Adjusted β**  **(99.9% CI)** | **Adjusted β**  **(99.9% CI)** | **Adjusted β**  **(99.9% CI)** |
| **Low**  (N=199) | -1.70  (-6.41, 3.0) | -0.39  (-5.73, 4.94) | -0.67  (-4.75, 3.40) | 2.23  (-1.97, 6.43) | 2.12  (-3.62, 7.88) |
| **Medium**  (N=1526) | -0.20  (-2.01, 1.59) | 0.54  (-1.37, 2.45) | -0.09  (-1.58, 1.39) | 0.01  (-1.25, 1.28) | 1.09  (-1.13, 3.32) |
| **High**  (N=1716) | -0.46  (-2.02, 1.09) | 0.35  (-1.44, 2.14) | -0.06  (-1.26, 1.13) | 0.67  (-0.32, 1.67) | 1.02  (-1.03, 3.09) |

^a^ Models are based on regressing the latent profiles of DM exposure on cognitive inflexibility on the same model, adjusting for basic confounders, including sex, continuous age, country of residence, total sleep duration, pubertal status, well-being score, in addition to media rules at home, being an only child, family structure and media multi-tasking (in categories). In all models, a random effect for family id was included, to consider family influences and to partially account for genetic factors influencing the cognitive functioning.

**Supplementary** **Table S8** shows the association between latent profiles of DM exposure and decision-making ability of children and adolescents, stratified by parental education attainment. The fully adjusted results indicate that children with high DM use except smartphone and who had parents with low educational attainment, showed an almost 3-point lower score for decision-making ability (β,-2.72, 99.9%CI, -15.1, 9.70), compared to children with low use of all media. In contrast, this association was observed to be positive among children and adolescents of parents with medium and high educational attainment. Children with high smartphone/internet, but medium TV/low PC use, and who had parents of medium and high educational attainment showed a 1.6 and 1.8 lower score for decision-making ability, respectively, compared to children with low use of all media. Nevertheless, these associations were not statistically significant.

**Supplementary** **Table S8. The association of latent profiles of digital media exposure with decision-making ability in European children and adolescents, by parental education attainment**

|  | **Media use profiles ^a^**  **(**Ref: Low DM use**)** | | | **Media multi-tasking**  **(**Ref: No MMT**)** | |
| --- | --- | --- | --- | --- | --- |
|  | **High DM use, except smartphone** | **High smartphone/internet, medium TV/low PC** | **Medium TV/Internet, low smartphone/PC** | **1-2 MMT** | **>2 MMT** |
| **Parental education attainment** | **Adjusted β**  **(99.9% CI)** | **Adjusted β**  **(99.9% CI)** | **Adjusted β**  **(99.9% CI)** | **Adjusted β**  **(99.9% CI)** | **Adjusted β**  **(99.9% CI)** |
| **Low**  (N=244) | -2.72  (-15.1, 9.70) | 0.82  (-12.4, 14.0) | -0.11  (-9.62, 9.38) | -3.33  (-11.8, 5.2) | -5.6  (-18.1, 6.95) |
| **Medium**  (N=1811) | 1.30  (-3.8, 6.41) | -1.57  (-6.98, 3.82) | -0.75  (-5.07, 3.56) | -0.73  (-4.21, 2.74) | -1.56  (-8.02, 4.89) |
| **High**  (N=1991) | 1.93  (-3.25, 7.11) | -1.88  (-7.68, 3.9) | -1.11  (-4.96, 2.73) | -1.59  (-4.78, 1.58) | -0.43  (-7.09, 6.21) |

^a^ Models are based on regressing the latent profiles of DM exposure on decision-making on the same model, adjusting for basic confounders, including sex, continuous age, country of residence, total sleep duration, pubertal status, well-being score, in addition to media rules at home, being an only child, family structure and media multi-tasking (in categories). In all models, a random effect for family id was included, to consider family influences and to partially account for genetic factors influencing the cognitive function.

**Supplementary** **References**

1 Rideout V. et al. Generation M2: Media in the lives of 8- to 18-year-olds, <<https://www.kff.org/other/poll-finding/report-generation-m2-media-in-the-lives/>> (2010).

2 UNESCO. International Standard Classification of Education. 2012 [cited 27.02.2023]; Available from: <http://uis.unesco.org/sites/default/files/documents/international-standard-classification-of-education-isced-2011-en.pdf>.

3 van Jaarsveld, C. H., Fidler, J. A., Simon, A. E. & Wardle, J. Persistent impact of pubertal timing on trends in smoking, food choice, activity, and stress in adolescence. *Psychosom Med* **69**, 798-806, doi:10.1097/PSY.0b013e3181576106 (2007).

4 Carskadon, M. A. & Acebo, C. A self-administered rating scale for pubertal development. *Journal of Adolescent Health* **14**, 190-195, doi:<https://doi.org/10.1016/1054-139X(93)90004-9> (1993).

5 Tanner, J. M. Growth and maturation during adolescence. *Nutrition Reviews* **39**, 43-55, doi:10.1111/j.1753-4887.1981.tb06734.x (1981).

6 Bullinger, M., Brütt, A. L., Erhart, M. & Ravens-Sieberer, U. Psychometric properties of the KINDL-R questionnaire: results of the BELLA study. *Eur Child Adolesc Psychiatry* **17 Suppl 1**, 125-132, doi:10.1007/s00787-008-1014-z (2008).

7 Cole, T. J. & Lobstein, T. Extended international (IOTF) body mass index cut-offs for thinness, overweight and obesity. *Pediatric Obesity* **7**, 284-294, doi:<https://doi.org/10.1111/j.2047-6310.2012.00064.x> (2012).

8 Bogl, L.-H., Kaprio, J., Brünings-Kuppe, C., Lissner, L. & Ahrens, W. in *Instruments for Health Surveys in Children and Adolescents* 291-301 (Springer, 2019).

9 Trent, K. & Spitze, G. D. Growing up without siblings and adult sociability behaviors. *J Fam Issues* **32**, 1178-1204, doi:10.1177/0192513X11398945 (2011).

10 Yang, J. *et al.* Only-child and non-only-child exhibit differences in creativity and agreeableness: evidence from behavioral and anatomical structural studies. *Brain Imaging and Behavior* **11**, 493-502, doi:10.1007/s11682-016-9530-9 (2017).

11 Amato, P. R. The impact of family formation change on the cognitive, social, and emotional well-being of the next generation. *Future Child* **15**, 75-96, doi:10.1353/foc.2005.0012 (2005).

12 Stahlmann, K. *et al.* A cross-sectional study of obesogenic behaviours and family rules according to family structure in European children. *Int. J. Behav. Nutr. Phys. Act.* **17**, 32, doi:10.1186/s12966-020-00939-2 (2020).
